# Factors Associated with Response to SGLT-2 Inhibitors and GLP-1 Receptor Agonists in Veterans with Type 2 Diabetes Mellitus †

**DOI:** 10.3390/jcm14124092

**Published:** 2025-06-10

**Authors:** Gunjan Arora, Sulman Hashmi, Samson Kaeli, Sarah Azad, Jaskaran Batra, Vijaya Deepika Perugu, Clifton Davis, Cyrus V. Desouza

**Affiliations:** 1 Department of Endocrinology, Omaha Nebraska VA Medical Center, Omaha, NE 68105, USA; garora@unmc.edu (G.A.); shashmi@unmc.edu (S.H.); sazad@unmc.edu (S.A.); jbatra@unmc.edu (J.B.); vperugu@unmc.edu (V.D.P.); clifton.davis@unmc.edu (C.D.); 2 Department of Endocrinology, University of Nebraska Medical Center, Omaha, NE 68198, USA; kksamson@unmc.edu

**Keywords:** type 2 diabetes mellitus, SGLT-2 inhibitors, GLP-1 receptor agonists, A1c, weight loss

## Abstract

**Background:** SGLT2 inhibitors (SGLT-2i) and GLP1 receptor agonists (GLP-1 RA) are recommended as the first line therapy for the management of type 2 diabetes mellitus (T2DM), particularly in patients with chronic kidney disease (CKD), cardiovascular disease (CVD), and heart failure (HF). Despite their established efficacy, there is limited evidence available to predict which subset of patients will respond favorably to them. We conducted this study to identify baseline characteristics to predict the response to therapy with SGLT-2i and GLP-1 RA. **Methods:** A retrospective analysis of the medical records was conducted at the Veteran Affairs Medical Center (VAMC) in Omaha, Nebraska, USA. Veterans who had completed 6–12 months of treatment with SGLT-2i or GLP-1 RA were included. Favorable treatment outcomes were a ≥0.5% reduction in glycosylated hemoglobin (HbA1c) or a ≥5% reduction in body weight; and those who achieved both outcomes were classified as adequate responders. **Results:** Patients in the GLP-1 RA group had 2.11 (95% CI: 1.45, 3.07) times the odds of achieving an adequate response as compared to patients in the SGLT-2i group in the unadjusted analysis, *p* < 0.001. HbA1c > 8% and older age was significantly associated with achieving an adequate response. **Conclusions:** Treatment with GLP-1 RA should be considered in Veterans with these characteristics.

## 1. Introduction

T2DM is the fastest growing global public health concern [1]. In the United States alone, approximately 30.3 million people—or 9.4% of the population—are diagnosed with diabetes, and the number is rising according to the Centers for Disease Control and Prevention. The incidence and prevalence of T2DM in the United States continues to rise [2]. It is estimated that the prevalence of diabetes (type 2 diabetes and type 1 diabetes) will increase by 54% to more than 54.9 million Americans between 2015 and 2030; annual deaths attributed to diabetes will climb by 38% to 385,800; and total annual medical and societal costs related to diabetes will increase 53% to more than $622 billion by 2030 [2].

Metformin, a biguanide, used to be the only initial pharmacological therapy recommended by the American Diabetes Association (ADA) for T2DM until the advent of GLP-1 RA, approved by the FDA for treatment of T2DM in 2005 [3] and SGLT-2i, approved by the FDA for treatment of T2DM in 2013 [4]. Metformin has a track-record of reducing HbA1c, safety and tolerability, widespread availability, and low cost, which makes it an ideal choice; however, a single agent is often not enough to lower a patient’s blood glucose level to the target range [5]. Numerous agents as second line options provide patients with the benefit of glucose reduction; however, many options, including sulfonylureas, thiazolidinediones, and insulin, have undesirable effects, such as weight gain and hypoglycemia, which potentially limit their overall utilization in certain patient populations. GLP-1 RA and SGLT-2i offer an innovative and unique advancement in the treatment of T2DM by not only benefiting blood glucose control but also preventing, stabilizing, and improving other diabetes-related complications, including but not limited to, cardiovascular events, nephropathy, obesity, etc. [5,6,7,8,9,10].

In 2022, the American Diabetes Association (ADA) updated their guidelines, recommending GLP-1 RA and SGLT-2i as first-line therapy for T2DM, especially for individuals with CVD, CKD, and HF [11]. While both GLP-1 RA and SGLT-2i have proven benefits for all of the conditions mentioned above, there are distinct differences between them—in their mechanism of action, pharmacology, treatment efficacy, adverse reactions, and limitations. Their effects have not been directly compared thus far, so which populations might benefit the most with either of these agents remains yet to be determined.

GLP-1 RA are a class of medications that work by mimicking the effects of the naturally occurring hormone GLP-1 to promote satiety and regulate blood glucose, leading to diabetes management and weight loss [12]. They facilitate pancreatic insulin secretion and suppress the release of glucagon in addition to suppressing hunger and delaying gastric emptying. Currently, 10 drugs under the class of GLP-1 RA medications have been approved by the FDA for the management of T2DM [12]. Most are administered as subcutaneous injections but there are some oral formulations. GLP-1 RA have demonstrated significant cardiovascular protective effects, reducing the risk of major adverse cardiovascular events (MACE) in individuals with or at high risk for atherosclerotic cardiovascular disease (ASCVD), including those with T2DM [13]. They have shown promise in slowing CKD progression in people with T2DM, particularly by reducing albuminuria and improving cardiovascular outcome [13]. Research studies have shown that GLP-1 RA appears to prevent hospitalizations for new onset heart failure and reduce symptoms in heart failure with preserved ejection fraction, thus, making them a strategic tool in management of heart failure [13].

In a recent meta-analysis, which incorporated SOUL and FLOW research studies, injectable and oral long-acting GLP-1 RA were shown to reduce the incidence of MACE and all the subcomponents by a broadly similar amount as well as that of all-cause mortality, heart failure hospitalization, and kidney events [14]. The remarkable consistency in the impact of GLP-1RA on the incidence of MACE and MACE subcomponents (including CV mortality) suggests a widespread antiatherosclerosis impact of these drugs that influences those and other processes that may culminate in CV death [14]. Unlike statins, GLP-1 RA not only exhibit CV benefits but also improve CKD outcomes, reduce the incidence of HHF, and lower all-cause mortality—reflecting distinct effects at multiple organ levels [14].

Their adverse effects include the more common gastrointestinal symptoms—nausea, vomiting, abdominal bloating, constipation, diarrhea—and the less common—pancreatitis, gastroparesis, bowel obstruction, and gall stones [13]. Their use is contraindicated with a history of pancreatitis, personal or family history of Multiple Endocrine Neoplasia type 2, severe gastrointestinal disease (gastroparesis or inflammatory bowel disease), active gall stone disease, severe chronic kidney disease, severe diabetic retinopathy, and pregnancy [13].

SGLT-2i are a class of medications that exert their effects by blocking the re-absorption of glucose in the kidneys, leading to increased glucose excretion, an effect independent of insulin, which is associated with modest weight loss [15]. The 2021 guidelines from the European Society of Cardiology (ESC) recommend SGLT2i as one of the four cornerstone therapies for patients with heart failure with reduced ejection fraction, and for patients with ejection fractions greater than 40%, SGLT2i receive a Class IA recommendation [16]. Additionally, the ESC 2023 guidelines for diabetes recommend SGLT2i (canagliflozin, empagliflozin, or dapagliflozin) for patients with type 2 diabetes and chronic kidney disease (CKD) with an estimated glomerular filtration rate (eGFR) ≥ 20 mL/min/1.73 m^2^, aiming to reduce cardiovascular risk and kidney failure with a Class IA recommendation [16]. SGLT-2i have proven benefits of a lower incidence of CVD, CV death, MACE, and all-cause mortality [17].

The pleotropic effects of SGLT-2i are wide spanning and becoming increasingly recognized. They have shown therapeutic benefits across multiple organ systems and acute and chronic conditions [18]. They facilitate endothelial functioning, nutrient signaling, and have anti-inflammatory properties, benefiting the management of coronary syndromes, arrythmias, cardiomyopathies, and cerebrovascular disease [18].

There are four FDA agents in this class of medications approved for the treatment of T2DM [15]. They are available as oral and pill formulations. They are contraindicated in pregnancy, breast feeding, and severe kidney impairment [15].

It would be of tremendous benefit to determine if an individual patient possesses certain characteristics that would confer a greater response to either SGLT-2i or GLP-1 RA before treatment initiation. Additionally, there is no head-to-head comparison conducted between these two classes of medications. We aim to conduct just that with an emphasis on diabetes control and weight loss. With this in mind, we conducted a retrospective analysis to guide clinicians, enhance therapeutic benefit derived from both agents, and streamline treatment protocols to off-set cost and medication shortages.

This study was conducted in a Veteran population, known to have higher cardiovascular disease, body mass index, and mental health illnesses necessitating the use of anti-psychotic medications compared to the general population, all of which may be associated with a worse metabolic profile. The efficacy of these two drugs may, therefore, have a different profile in this special population.

## 2. Materials and Methods

### 2.1. Study Design

The medical records for all Veterans with T2DM managed with any GLP-1RA medication or SGLT-2i medication at the Omaha, Nebraska Veteran Affairs (VA) Medical Center from 1 January 2015 to 31 December 2022 were retrospectively reviewed following Institutional Review Board (IRB) approval. The study was conducted according to the guidelines of and approved by NWIHCS VA Research and Development Committee (R&DC) of institution Nebraska-Western Iowa VA Health Care System (Protocol title 1581616-13 and date of approval 10/5/2020).

Electronic medical records were accessed by the members of the research team to screen and collect research data about the participants in the study. No patient identifiers were disclosed to anyone outside of the approved research team. No paper records containing patient identifiers were maintained. Electronic patient health information (PHI) was stored in the research drive on the VA server.

### 2.2. Study Population Selection

Separate lists of Veterans with T2DM who were initiated on treatment with a SGLT-2i and GLP-1 RA between 1 January 2015 and 31 December 2022 were generated. We chose to start our inclusion from the year 2015 because by 2015, both GLP-1 RA and SGLT-2i were approved by the FDA for the treatment of T2DM (GLP-RA were first approved by the FDA for the treatment of TDM in 2005 and SGLT-2i were first approved by the FDA for the treatment of T2DM in 2013) and were becoming increasingly utilized for the same. EMR charts were reviewed by the members of the research team. Patients that had completed at least 6 months and up to 12 months of treatment with either medication underwent further chart review.

Baseline characteristics were then assessed, comprising age, sex, race, comorbidities, weight, BMI, HbA1c, eGFR, blood pressure, chemistry panel, and lipid panel. Age < 19 years or more than 89 years, diagnosis of type 1 diabetes, estimated baseline eGFR of <30 mL/minute/1.73 m^2^, history of a malignant neoplasm within the prior 5 years (except for basal cell carcinoma, squamous cell carcinoma, and carcinoma-in-situ) and those who did not have key data documented (age, sex, race, other diabetes medications, baseline weight, height, baseline HbA1c, BP measurement, serum chemistry panel, and serum lipid panel) were excluded.

### 2.3. Inclusion and Exclusion Criteria Are Summarized Below

Inclusion criteria (must meet all three):

1. Diagnosis of T2DM;
2. Age 19–89 years;
3. On treatment with a GLP-1 RA or SGLT-2i for at least 6 months and up to 12 months.

Exclusion criteria (any):

1. Diagnosis of type 1 diabetes mellitus;
2. Estimated baseline eGFR of <30 mL/minute/1.73 m^2^.

History of a malignant neoplasm within the past 5 years except for basal cell carcinoma, squamous cell skin cancer, and carcinoma in-situ.

Patients who did not have documentation of all key data: age, sex, race, other diabetes medications, baseline weight, height, baseline HbA1c, BP measurement, serum chemistry panel, and serum lipid panel. Patients who did not have documentation of all key data were excluded with case-wide deletion in the final analysis.

The demographic information was collected nearest to the start of treatment with either a GLP-1 RA or SGLT-2i. For every patient in the study, treatment progress was recorded for a minimum of 6 months by recording changes in HbA1c, body weight/BMI, diabetes regimen, GFR, and BP. We also monitored for changes in co-morbid conditions. In addition, we collected data on adverse drug reactions specific to both GLP-1 RA and SGLT-2i as noted below:

For GLP-1 RA adverse reaction data collected: nausea, vomiting, diarrhea, constipation, and pancreatitis
For SGLT-2i adverse reaction data collected: urinary tract infections, genitourinary fungal infection, dyslipidemia, nausea, acute renal failure, and ketoacidosis.

Once the data collection was completed, identifiers were removed, and the dataset was coded numerically. The de-identified data was archived in the study folder on the VA server for the research team to access.

**Figure 1:**
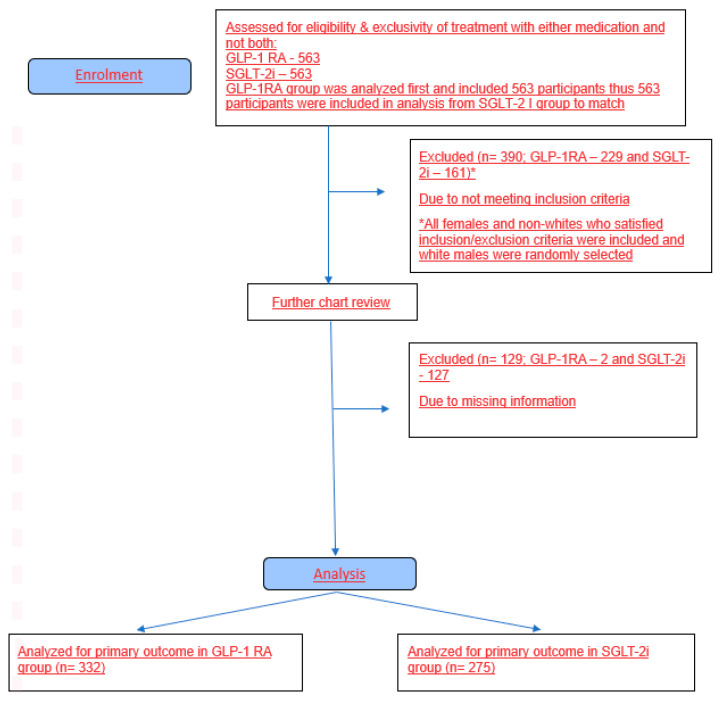
Consort flow diagram.

Due to the low number of female and non-white patients in the sample, all females and non-white patients who met the inclusion criteria were included in the analysis; and white males who met the inclusion criteria were randomly selected from the remaining patients to meet the desired sample size. Our aim was to include 600 patients in our study, approximately 300 patients on GLP-1RA and 300 patients on SGLT-2i for T2DM management. The study flow chart is summarized in a consort diagram (Figure 1) at the end of this section.

### 2.4. Study Outcomes

The two main outcomes were a ≥0.5% reduction in glycosylated hemoglobin (HbA1c) and a ≥5% reduction in pre-treatment body weight.

An adequate response was defined as having both the treatment response thresholds met.

### 2.5. Statistical Analysis

Counts and percentages were calculated for each specific outcome. Descriptive statistics for continuous data were given as means and standard deviations. Associations between the complete response variable and categorical variables of interest were assessed using Chi-Square tests. Independent samples t-tests were used to examine differences in the means of variables of interest between the complete response groups. A logistic regression was run with complete response as the dichotomous outcome and included all variables that had a bivariate *p*-value with the outcome variable less than 0.20, in addition to other variables which were chosen for inclusion a priori for their clinical relevance. For categorical variables with more than two groups, 95% confidence intervals (CI) were Bonferroni-adjusted. All analyses were performed using SAS software version 9.4 (SAS Institute Inc., Cary, NC, USA).

## 3. Results

Out of a cohort of 866 patients, 607 met the inclusion criteria (332 in the GLP-1 RA group and 275 in the SGLT-2i group).

### 3.1. Subsection: Baseline Characteristics and Results

Demographic and Baseline Clinical Characteristics of Study Sample

As shown in Table 1, our study population comprised a total of 607 patients: 54.7% [*n* = 332] in the GLP-1 RA and 45.3% [*n* = 275] in the SGLT-2i group. There was an under-representation of females at 8.7% [*n* = 53] and non-white races at 11.5% [*n* = 70]. The mean age of participants was 67.4 years. Additionally, 66.9% [*n* = 406] of participants had a baseline HbA1c of 8% or higher and 78.0% [*n* = 472] had a baseline BMI of 30 or more. Furthermore, 48.1% [*n* = 292] were not using insulin while 34.9% [*n* = 212] were using less than 100 units of insulin per day and 17.0% [*n* = 103] were using more than 100 units of insulin per day. Metformin use was seen in 77.3% [*n* = 464] of participants while data was missing for seven patients.

### 3.2. Results: A1c Reduction, Weight Loss, and Bivariate Analysis of Baseline and Clinical Characteristics by Adequate Response

As shown in Table 2, 72.6% of patients in GLP-1 RA group achieved an A1c reduction of ≥0.5% from baseline compared to 45.5% of patients in SGLT-2i group, *p* < 0.001. Additionally, 47.6% of patients in the GLP-1 RA group achieved weight loss of ≥5% from baseline compared to 41.5% in the SGLT-2i group, *p* = 0.13; however, this was not statistically significant.

Bivariate analysis showed that there was a higher proportion of patients in the GLP-1 RA group who achieved an adequate response (34%) compared to patients in the SGLT-2i group (19.6%), (*p* < 0.001). The patients with an HbA1c of 8% or higher had a higher proportion of those who achieved an adequate response (33%) compared to patients who had a baseline HbA1c of less than 8% (16.4%), (*p* < 0.001). Patients with a BMI ≥ 30 had a higher proportion of those who achieved an adequate response (30.1%) compared to those with BMI < 30 (18.8%), (*p* = 0.01).

There was no statistically significant difference noted in the response achieved in regard to the use of insulin or Metformin. 24.7% of patients not on insulin achieved an adequate response as compared to 27.8% of patients on less than 100 units of insulin per day and 35% of patients on more than 100 units of insulin per day, (*p* = 0.13); and 28.4% of patients on Metformin achieved an adequate response compared to 25% not on Metformin, (*p* = 0.43).

Differences in age and sex were not statistically significant between those who achieved an adequate response and those who did not. Additionally, the differences in systolic blood pressure and triglyceride levels were not statistically significant between the adequate responders and non-responders.

### 3.3. Results: Logistic Regression Model Results with Adequate Response as the Outcome

Table 3 shows logistic regression model results with adequate response as the outcome. Adequate response was defined as meeting desired outcome criteria for both weight loss and HbA1c reduction. The variables described in the table were selected based on clinical judgement: age (in decades), sex, race, baseline HbA1c, baseline BMI, baseline systolic BP, baseline triglyceride, baseline insulin use, and baseline Metformin use. Patients in the GLP-1 RA group had 2.11 (95% CI: 1.45, 3.07) times the odds of achieving an adequate response compared to patients in SGLT-2i group in the unadjusted analysis, *p* < 0.001 and 1.86 (95% CI: 1.14, 3.03) times the odds of achieving an adequate response in the adjusted analysis, *p* = 0.01 (adjusted for age, sex, race, baseline HbA1c, baseline BMI, baseline systolic BP, baseline TG level, baseline insulin use, and baseline Metformin use). The association between the GLP-1 RA group and achievement of an adequate response remained statistically significant even after adjusting for variables of clinical interest. Variables associated with an adequate response, which were found to be statistically significant, include the following: older age with odds ratio of 1.31 (95% CI: 1.03, 1.68), *p* = 0.03 and a baseline HbA1c of 8.0% or higher with odds ratio of 2.33 (95% CI: 1.44, 3.76), *p* = 0.00

## 4. Discussion

This study was performed with Veterans who have a disproportionately high incidence of type 2 diabetes and its resultant complications. There are various factors that might contribute to this, including a much greater use of antipsychotics and higher BMI than the general population. The comparative effectiveness of the use of GLP-1 RA vs. SGLT2-i has not been exclusively studied in this population.

Our study shows that there is a greater proportion of Veterans who achieved a clinically significant A1c reduction with GLP-1 RA than SGLT2-i. This is in line with previous studies in the general population. However, our study showed that the weight loss achieved by Veterans on GLP-1 RA was not very different from SGLT2-i and the percentage in weight reduction was comparatively small. This is very different from the general population where GLP-1 RA have been shown to be much more efficacious in terms of weight loss. The STEP (Semaglutide Treatment Effect in People with Obesity) program, a series of clinical trials, demonstrated in the STEP 2 trial that participants with type 2 diabetes and overweight or obesity experienced a mean weight loss of 9.6% when taking Semaglutide 2.4 mg once weekly, along with a lifestyle intervention, compared to 3.4% with lifestyle intervention alone [19].

The GRADE (Glycemia Reduction Approaches in Diabetes: A Comparative Effectiveness Study) trial found that the participants in the liraglutide group experienced significantly more weight loss over the 4-year study period compared to those in the Glargine, Glimepiride, and Sitagliptin groups [20]. We postulate that the discrepancy between the weight loss achieved in our study population and the general population and trials, such as STEP and GRADE, might be due to our population having a greater use of older antipsychotic medications (which contribute to metabolic derangements) as well as less use of the highest dose of Semaglutide (a GLP-1 RA) at 2 mg weekly as 1 mg weekly is the maximum allowed dose at the VA due to shortage. The 1 mg dose may not have been effectual for weight loss in this population whereas higher doses might have been. Medication adherence, prescription coverage variability, and inadvertent lapses in between filing prescriptions may be additional factors at play; however, these factors, at least to a certain degree, are present in the general population as well.

Furthermore, more efficacious GLP-1 RA’s, such as Tirzepatide are highly restricted at our VA. The SURPASS trials showed Tirzepatide, a dual GIP/GLP-1 receptor agonist, led to clinically significant weight loss in individuals with type 2 diabetes with an average weight reduction of 7–14% of body weight and a substantial proportion of participants achieving weight reductions of 15% or more [21].

In our study, an adequate response defined as a significant reduction in A1c and weight was much more likely to occur in Veterans on a GLP-1 RA than SGLT-2i. Factors associated with an adequate response were higher baseline A1c, BMI ≥ 30, and older age. These factors would be expected to be linked to better outcomes as shown in previous studies.

There are limitations to interpreting this study as it is retrospective. However, it spanned a long duration of time, and the numbers are substantial. We assessed the two main outcomes for the participants, not at a fixed point in time after treatment initiation with GLP-1 RA or SGLT-2i, but rather at a range of 6–12 months post treatment initiation with either agent. This decision was made because this study is a retrospective analysis and so a time frame allows for more flexibility and a bigger sample size. Consistent with the VA population, the percentage of females was low in this study.

In conclusion, GLP-1 RA had a nearly two-fold likelihood of having an adequate response after a 6–12 months treatment period compared to the SGLT-2i group in the Veteran population. Higher baseline A1c, BMI ≥ 30, and older age were strong predictors of an adequate response, whereas other baseline variables were not significantly associated with the outcome. Treatment with GLP-1 RA should be considered in Veterans with these characteristics.

## Figures and Tables

**Table 1:** Demographic and baseline clinical characteristics of study population.

	Total (*n* = 607)
**Medication**	*n* (%)
GLP-1 RA	332 (54.7%)
SGLT-2i	275 (45.3%)
**Age**	
Mean (SD)	67.4 (9.67)
*n*	607
**Sex**	
Female	53 (8.7%)
Male	554 (91.3%)
**Race**	
Black	56 (9.2%)
Other	14 (2.3%)
White	537 (88.5%)
**HbA1c**	
<8.0%	201 (33.1%)
8.0% or higher	406 (66.9%)
**BMI**	
<30	133 (22.0%)
30 or higher	472 (78.0%)
missing	2
**Systolic BP**	
Mean (SD)	131.5 (18.33)
*n*	606
**TG**	
Mean (SD)	228.1 (142.65)
*n*	565
**Insulin use per day**	
None	292 (48.1%)
100+ units	103 (17.0%)
<100	212 (34.9%)
**Metformin use**	
No	136 (22.7%)
Yes	464 (77.3%)
missing	7

**Table 2:** Bivariate analysis of demographic and clinical characteristics by adequate response.

	Adequate Response (Both Weight Reduction and A1c Reduction)		
	No (*n* = 440)	Yes (*n* = 167)	*p*-Value
**Medication**, *n* (%)			<0.001
GLP-1RA	219 (66.0%)	113 (34.0%)	
SGLT-2i	221 (80.4%)	54 (19.6%)	
**Age**			0.40 ^†^
Mean (SD)	67.2 (9.54)	67.9 (9.99)	
*n*	440	167	
**Sex**, *n* (%)			0.89
Female	38 (71.7%)	15 (28.3%)	
Male	402 (72.6%)	152 (27.4%)	
**Race**, *n* (%)			0.34
Black	43 (76.8%)	13 (23.2%)	
Other	8 (57.1%)	6 (42.9%)	
White	389 (72.4%)	148 (27.6%)	
**HbA1c**, *n* (%)			<0.001
<8.0	168 (83.6%)	33 (16.4%)	
8.0 or higher	272 (67.0%)	134 (33.0%)	
**BMI**, *n* (%)			0.01
<30	108 (81.2%)	25 (18.8%)	
30 or higher	330 (69.9%)	142 (30.1%)	
*n*	2	0	
**Systolic BP**			0.43 ^†^
Mean (SD)	131.1 (18.69)	132.4 (17.37)	
*n*	440	166	
**TG**			0.61 ^†^
Mean (SD)	226.2 (147.29)	233.0 (129.90)	
*n*	410	155	
**Insulin**			0.13
None	220 (75.3%)	72 (24.7%)	
100+	67 (65.0%)	36 (35.0%)	
<100	153 (72.2%)	59 (27.8%)	
**Metformin**, *n* (%)			0.43
No	102 (75.0%)	34 (25.0%)	
Yes	332 (71.6%)	132 (28.4%)	
Missing	6	1	

*p*-values from Chi-Square tests unless otherwise indicated. ^†^ *p*-value from Independent Samples *t*-test.

**Table 3:** Logistic regression model results with adequate response as the outcome.

Unadjusted Analysis						Adjusted Analysis				
	Reference	Unadjusted Odds Ratio	95% Confidence Interval		*p*-Value	Reference	Adjusted Odds Ratio	95% Confidence Interval		*p*-Value
**Medication**										
GLP-1RA	SGLT-2i	2.11	1.45	3.07	<0.001	SGLT-2i	1.86	1.14	3.03	0.01
**Age (in decades)**						None	1.31	1.03	1.68	0.03
**Sex**										
Female						Male	1.67	0.82	3.43	0.16
**Race**										
Black						White	0.91	0.39	2.13	0.44
Other						White	2.29	0.46	11.48	0.44
**Baseline HbA1c**										
8.0 or higher						<8.0	2.33	1.44	3.76	0.001
**Baseline BMI**										
30 or higher						<30	1.51	0.87	2.64	0.15
**Baseline Systolic BP**							1.01	0.91	1.12	0.93
**Baseline TG**							1.00	0.99	1.02	0.65
**Baseline Insulin use**										
100+						Not on insulin	1.09	0.53	2.25	0.88
<100						Not on insulin	0.95	0.53	1.72	0.88
**Baseline** **Metformin use**										
Yes						Not on Metformin	1.54	0.95	2.52	0.08

Reference Confidence intervals for variables with more than two groups are Bonferroni adjusted.

## Data Availability

The datasets generated during and/or analyzed during the current study are not publicly available as they pertain to the VA population.

## References

[1] Hossain M.J., Al-Mamun M., Islam M.R. (2024). Diabetes mellitus, the fastest growing global public health concern: Early detection should be focused. Health Sci. Rep..

[2] Rowley W.R., Bezold C., Arikan Y., Byrne E., Krohe S. (2017). Diabetes 2030: Insights from Yesterday, Today and Future Trends. Popul. Health Manag..

[3] Latif W., Lambrinos K.J., Patel P., Rodriguez R. Compare and Contrast the Glucagon-Like Peptide 1 Receptor Agonists (GLP1RAs). https://www.ncbi.nlm.nih.gov/books/NBK572151/.

[4] National Institute of Diabetes and Digestive and Kidney Disease Story of Discovery: SGLT2 Inhibitors: Harnessing the Kidneys to Help Treat Diabetes. https://www.niddk.nih.gov/news/archive/2016/story-discovery-sglt2-inhibitors-harnessing-kidneys-help-treat-diabetes#:~:text=The%20first%20SGLT2%20inhibitor%20to,Jardiance%C2%AE)%20in%20August%202014.

[5] ElSayed N.A., Aleppo G., Aroda V.R., Bannuru R.R., Brown F.M., Bruemmer D., Collins B.S., Hilliard M.E., Isaacs D., Johnson E.L. (2023). 9. Pharmacologic Approaches to Glycemic Treatment: Standards of Care in Diabetes—2023. Diabetes Care.

[6] Prasad-Reddy L., Isaacs D. (2015). A clinical review of GLP-1 receptor agonists: Efficacy and safety in diabetes and beyond. Drugs Context.

[7] Tariq S., Ali M.A., Hassan Iftikhar H.M., Fareh Ali M., Shah S.Q.A., Perveen F., Zaman T. (2024). Long-Term Cardiovascular Outcomes of Glucagon-Like Peptide-1 (GLP-1) Receptor Agonists in Type 2 Diabetes: A Systematic Review. Cureus.

[8] Pan H.C., Chen J.Y., Chen H.Y., Yeh F.Y., Sun C.Y., Huang T.T.M., Wu V.C. (2024). GLP-1 receptor agonists’ impact on cardio-renal outcomes and mortality in T2D with acute kidney disease. Nat. Commun..

[9] McGuire D.K., Shih W.J., Cosentino F., Charbonnel B., Cherney D.Z.I., Dagogo-Jack S., Pratley R., Greenberg M., Wang S., Huyck S. (2021). Association of SGLT2 Inhibitors With Cardiovascular and Kidney Outcomes in Patients With Type 2 Diabetes: A Meta-analysis. JAMA Cardiol..

[10] Rabizadeh S., Nakhjavani M., Esteghamati A. (2019). Cardiovascular and Renal Benefits of SGLT2 Inhibitors: A Narrative Review. Int. J. Endocrinol. Metab..

[11] American Diabetes Association Professional Practice Committee (2025). Pharmacological Approaches to Glycemic Treatment: Standards of Care in Diabetes—2025. Diabetes Care.

[12] Dungan K., DeSantis A. (2024). Glucagon-Like Peptide 1-Based Therapies for the Treatment of Type 2 Diabetes Mellitus. UpToDate. https://www.uptodate.com/contents/glucagon-like-peptide-1-based-therapies-for-the-treatment-of-type-2-diabetes-mellitus.

[13] Marx N., Husain M., Lehrke M., Verma S., Sattar N. (2022). GLP-1 Receptor Agonists for the Reduction of Atherosclerotic Cardiovascular Risk in Patients With Type 2 Diabetes. Circulation.

[14] Lee M.M.Y., Sattar N., Pop-Busui R., Deanfield J., Emerson S.S., Inzucchi S.E., Mann J.F.E., Marx N., Mulvagh S.L., Poulter N.R. (2025). Cardiovascular and Kidney Outcomes and Mortality With Long-Acting Injectable and Oral Glucagon-Like Peptide 1 Receptor Agonists in Individuals With Type 2 Diabetes: A Systematic Review and Meta-analysis of Randomized Trials. Diabetes Care.

[15] Padda I.S., Mahtani A.U., Parmar M. (2025). Sodium-Glucose Transport Protein 2 (SGLT2) Inhibitors. StatPearls.

[16] Cersosimo A., Drera A., Adamo M., Metra M., Vizzardi E. (2024). Exploring the Cardiorenal Benefits of SGLT2i: A Comprehensive Review. Kidney Dial..

[17] Mavrakanas T.A., Tsoukas M.A., Brophy J.M., Sharma A., Gariani K. (2023). SGLT-2 inhibitors improve cardiovascular and renal outcomes in patients with CKD: A systematic review and meta-analysis. Sci. Rep..

[18] Armillotta M., Angeli F., Paolisso P., Belmonte M., Raschi E., Di Dalmazi G., Amicone S., Canton L., Fedele D., Suma N. (2025). Cardiovascular therapeutic targets of sodium-glucose co-transporter 2 (SGLT2) inhibitors beyond heart failure. Pharmacol. Ther..

[19] Bergmann N.C., Davies M.J., Lingvay I., Knop F.K. (2023). Semaglutide for the treatment of overweight and obesity: A review. Diabetes Obes. Metab..

[20] Green J.B., Everett B.M., Ghosh A., Younes N., Krause-Steinrauf H., Barzilay J., Desouza C., Inzucchi S.E., Pokharel Y., Schade D. (2024). Cardiovascular Outcomes in GRADE (Glycemia Reduction Approaches in Type 2 Diabetes: A Comparative Effectiveness Study). Circulation.

[21] Jung H.N., Jung C.H. (2022). The Upcoming Weekly Tides (Semaglutide vs. Tirzepatide) against Obesity: STEP or SURPASS?. J. Obes. Metab. Syndr..

[22] Hashmi S., Samson K., Arora G., Azad S.K.H., Awad D., Desouza C.V. Factors associated with the response to SGLT2 inhibitors and GLP-1 Receptor Agonists in Veterans with Type 2 Diabetes. Proceedings of the Endocrine Society National Conference.

